# Physic at Oxford

**Published:** 1904-07-23

**Authors:** 


### PHYSIC AT OXFORD.

The meeting of the British Medical Association at Oxford serves to recall the debt which physic owes to the University. From time immemorial, that is to say, from the twelfth century onwards, medicine has been one of the three higher faculties which engaged the attention of those who had passed through the ordinary routine of University learning. Divinity and law must always have attracted the more ambitious minds, for then, as now, the prizes they offered were greater than could be obtained from the practice, whether speculative or actual, of the science of medicine. But in every college some of the fellowships were usually filled by graduates in medicine, and it was often customary to combine a degree in divinity with an M.D. or an M.B. Thus some of the earliest graduates of medicine like Dr. Gilbert Kymer, who served as Proctor, and was Chancellor in 1431, became Dean of Salisbury, though he continued to practise medicine, for in June 1455 he was summoned to Windsor to attend Henry VI. in the fit of imbecility which attacked him soon after the first battle of St. Albans. The introduction of Greek into England was largely due to the Oxford school of which Dr. Linacre was one of the most illustrious members, and to his action also the College of Physicians of London owes in great measure its foundation. But the real reputation of Oxford as a medical school depends upon the remarkable band of workers in the seventeenth century who established the Royal Society. These men represented all that is best in physic. They were in the first place, and before all things, investigators. The work of Willis and of Lower was a permanent and distinct advance in anatomy. Wren was so great an architect that his fame as Savilian Professor of Astronomy and as a demonstrator of anatomy is quite eclipsed. Scarborough and Millington represent the type of distinguished clinical physicians who made money, but to nothing like the same amount as did their more fortunate junior Radcliffe. The fame of Dr. Radcliffe survives by reason of his benefactions to the University, for the camera, the observatory, and the library are patent to everyone. This outburst of original work was very evanescent. Its beginning was coincident with, and perhaps in some measure due to, the residence of William Harvey at Merton. But the *virtuosi* soon moved back to London, and throughout the eighteenth century Oxford was barren from a medical standpoint. The reawakening took place in the middle of the last century. Acland, ably seconded by Rolleston and Dean Liddell, determined that the Oxford students of medicine should have a first-rate education in science and in all those subjects which are ancillary to medicine, for they recognised, clearly enough, that a town situated in the heart of an agricultural district could never be made a good clinical centre either for medicine or surgery. It was undesirable to establish a medical school which should compete with the licensing bodies then in existence, and it was equally undesirable to call into existence a large number of medical students who might possibly undermine the somewhat antiquated restrictions which the University still exercises over those *in statu pupillari*. Quantity, therefore, was deliberately sacrificed to quality. Undergraduates who desire to obtain the medical degrees of the University are placed upon exactly the same footing as those who intend to enter the Church, go to the Bar, or become schoolmasters. No college is especially connected with medicine, and every undergraduate must of necessity obtain a degree in Arts before he can be presented for the M.B. The result has been eminently satisfactory; for although the total number of medical graduates is well under 250, more than 60 have been elected to teaching posts in the University, or to hospitals in London or the provinces. They form, too, a very compact and well-disciplined body, as was shown recently when they protested almost unanimously against any undue haste in the election of a Regius Professor in succession to Sir John Burdon Sanderson. The protest was accepted, and the University will therefore be unable to receive the British Medical Association by its official medical head, the Regius Professor, though his place will be worthily filled by his substitute, Professor Arthur Thomson, the Professor of Anatomy, who has been appointed to perform the duties *ad interim*.

